# Patterned Piezoelectric Scaffolds for Osteogenic Differentiation

**DOI:** 10.3390/ijms21218352

**Published:** 2020-11-07

**Authors:** Teresa Marques-Almeida, Vanessa F. Cardoso, Miguel Gama, Senentxu Lanceros-Mendez, Clarisse Ribeiro

**Affiliations:** 1 CF-UM-UP, Centro de Física das Universidades do Minho e Porto, Campus de Gualtar, Universidade do Minho, 4710-057 Braga, Portugal; talmeida@fisica.uminho.pt (T.M.-A.); vanessa@dei.uminho.pt (V.F.C.); 2 CEB, Centro de Engenharia Biológica, Campus de Gualtar, Universidade do Minho, 4710-057 Braga, Portugal; fmgama@deb.uminho.pt; 3 CMEMS-UMinho, Campus de Azurém, Universidade do Minho, 4800-058 Guimarães, Portugal; 4 BCMaterials, Basque Center for Materials, Applications and Nanostructures, UPV/EHU Science Park, 48940 Leioa, Spain; 5 IKERBASQUE, Basque Foundation for Science, 48009 Bilbao, Spain

**Keywords:** piezoelectric, electroactive, patterning, cell differentiation, bone tissue engineering

## Abstract

The morphological clues of scaffolds can determine cell behavior and, therefore, the patterning of electroactive polymers can be a suitable strategy for bone tissue engineering. In this way, this work reports on the influence of poly(vinylidene fluoride-co-trifluoroethylene) (P(VDF-TrFE)) electroactive micropatterned scaffolds on the proliferation and differentiation of bone cells. For that, micropatterned P(VDF-TrFE) scaffolds were produced by lithography in the form of arrays of lines and hexagons and then tested for cell proliferation and differentiation of pre-osteoblast cell line. Results show that more anisotropic surface microstructures promote bone differentiation without the need of further biochemical stimulation. Thus, the combination of specific patterns with the inherent electroactivity of materials provides a promising platform for bone regeneration.

## 1. Introduction

Bone tissue regeneration represents one of the major challenges of biomedicine. As in other areas of biomedicine, efforts are being conducted on replacing conventional approaches with more biomimetic ones. In this scope, tissue-specific active scaffolds are being developed, combining stem or precursor cells and physic/chemical cues, that synergistically stimulate the repairing process, eventually being replaced by the patient’s own tissue [1].

Bone can be differentiated, according to the macrostructure, in trabecular (porous) and cortical (compact). At the cellular and molecular levels, bone is composed of cells (osteoblasts, osteoclasts, osteocytes, and bone lining cells) merged in a non-oriented collagen type I matrix, mineralized by hydroxyapatite (HA) that is responsible for toughening the bone [2]. When an injury takes place, a defective microenvironment compromises the normal resorption and regrowth of bone tissue, and consequently its regeneration.

Investigations are being developed based on different strategic cues, such as electromechanical [3,4], chemical [5,6], and morphological [7,8], in an attempt to recreate tissue-specific microenvironments and thus trigger their natural recovery. Morphological cues have been demonstrated to effectively influence cellular proliferation and differentiation, the cell–scaffold interaction triggering a series of physical-chemical reactions. Cells sense the site they are attached to and mechanically transduce that information (hardness, curvature, and shape) into morphological changes [9]. When favorable topographical signals are presented at the surface of the scaffold, they can trigger the initiation of mechanosensitive cell cascades and thus a cell’s differentiation signaling pathways [10,11,12]. However, the effective mechanism by which morphological cues regulate cell fate, in terms of orientation, morphology, proliferation, and differentiation, is still barely understood. Given the topographic complexity of its natural microenvironment, bone cells are adaptable to different scaffolds’ architectures, although it is known that their phenotype is not favored in aligned morphologies, unlike for instance in the case of myoblasts or neurons. Different structures have been developed to grow bone tissue, but only a few trials with micropatterned scaffolds have been reported so far. Micropatterned scaffolds based on polycaprolactone (PCL)/polylactic-co-glycolic acid (PLGA) have been applied for periodontal tissue regeneration [13], demonstrating that micropatterning can effectively enhance tissue responses. HA ceramics with surface micropatterning have been demonstrated to promote the osteogenic differentiation [14]. In addition to the influence of the morphology, studies have demonstrated that piezoelectric biomaterials, capable of providing mechano-electrical stimuli, can enhance bone cell differentiation and regeneration [15,16], as those electro active stimuli effectively mimic the natural cell’s microenvironment.

As previously shown [7], pre-osteoblast cells maintain their phenotype when adhered to a scaffold with isotropic hexagonal surface topography, unlike what happens in the linear topography. The influence of both morphologies, hexagonal and linear, on pre-osteoblasts proliferation and differentiation is here studied in an attempt to determine whether it is possible to physically induce the differentiation of bone precursor cells, avoiding the use of biochemical differentiation factors. In addition, it is known that bone tissue presents inherent piezoelectricity, and therefore, morphological features were patterned on a piezoelectric polymer, using the non-biodegradable polymer of poly(vinylidene fluoride-co-trifluoroethylene) (P(VDF-TrFE) once it presented the highest piezoelectric coefficient among all the polymers [17]. This was done to allow the development of electroactive platforms for bone tissue engineering that combines the morphology and the possibility of further mechano-electrical stimuli to the cells.

## 2. Results and Discussion

### 2.1. Cellular Proliferation

The preference of MC3T3-E1 cells for adhesion on surfaces with specific topographies has been previously reported [7] and was further assessed in the present study. Further, it is to notice that it is essential, from a molecular point of view, to obtain the materials in the electroactive phase, i.e., in the all-trans b-phase chain conformation, to provide electromechanical cues to the cells at the nano- [18] or microscale [15], depending on the poling state of the material [19]. It is confirmed that the scaffolds are obtained in the β-phase, identified by the vibration modes at ∼510, 840, 1287, and 1400 cm^−1^ [20], representing, respectively, the CF2 bending; CF2 symmetric stretching; CF2 and CC symmetric stretching and CCC bending; and CH2 wagging and CC antisymmetric stretching (see supplementary information Figure S1).

Proliferation assays were performed using control scaffolds (non-patterned) and patterned ones featuring topographies with 25, 75, and 150 µm wide lines or hexagons.

**Figure 1:**
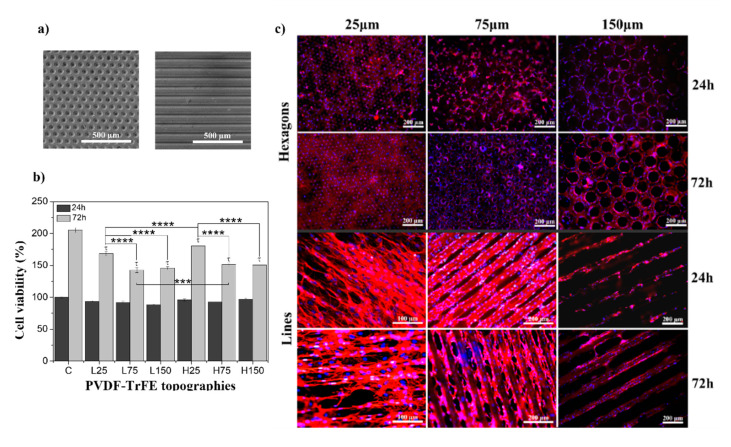
(**a**) SEM images of patterned poly(vinylidene fluoride-co-trifluoroethylene) (P(VDF-TrFE)) scaffolds of hexagons and lines microstructures with 75 μm dimensional features; (**b**) cellular viability obtained after MTS assay of MC3T3-E1 cells in contact with control; (**c**) representative images of pre-osteoblast culture on linear topographies with 25, 75, and 150 µm (L25, L75, and L150, respectively) and hexagonal topographies with 25, 75, and 150 µm (H25, H75 and H150, respectively), at 24 h and 72 h timepoints (nucleus stained with DAPI-blue and cytoskeleton stained with tetramethylrhodamine (TRITC)-red). ^τ^ *p* < 0.0005 vs. control; **** *p* < 0.0001, *** *p* < 0.001 (two-way ANOVA); (**c**) MC3T3-E1 adhesion on patterned P(VDF-TrFE) scaffolds with 25, 75, and 150 μm dimensions, in 24 and 72 h of contact.

For both linear and hexagonal topographies, the smaller lines and hexagons (25 µm) show higher cell viability than the larger ones (Figure 1a). Checking the immunofluorescence images (Figure 1b), it is observed that cells show, contrary to the non-patterned control samples (see supplementary information Figure S4), the preferential orientation of the microstructure of the patterned P(VDF-TrFE) scaffolds, being slightly lower with smaller features (25 µm). Thus, the perception of more cellular viability is provided by the fact that cells have more contact area to proliferate, since they adhere to all the scaffolds’ surfaces. Bone cells are quite resilient to different surroundings, since they are naturally present in different microenvironments. Therefore, MC3T3-E1 proliferate quite well in both topography types and dimensions, although significantly more on the isotropic hexagonal topography. Immunofluorescent images disclose the compromised cellular phenotype over linear topography, being elongated and not round as is common. Their elongated phenotype suggests that the normal fate of cells is being negatively influenced by this physical stimulus, contrary to the hexagonal topography. This may indicate that, in a differentiation phase, this stimulus may cause cells to be unable to differentiate, or to differentiate into an unwanted cell type.

### 2.2. Differentiation Assays

This study was performed in order to investigate the effect of the surface topography on the osteoblasts differentiation, using alkaline phosphatase (ALP) quantification assay as early marker, since it plays a critical role in bone formation [21,22], and alizarin red staining as late marker of osteoblast differentiation.

For the differentiation assays, control scaffolds (non-patterned) and patterned ones with 75 µm features (linear or hexagonal topographies) were tested. The differentiation in the two conditions was assessed in the absence (GM) and in the presence (DM) of the biochemical inducer for 21 days. The goal is to understand the physical effect, namely, the topography, of the patterned scaffolds on the cells’ differentiation fate, with or without the cells being exposed to biochemical inducers.

**Figure 2:**
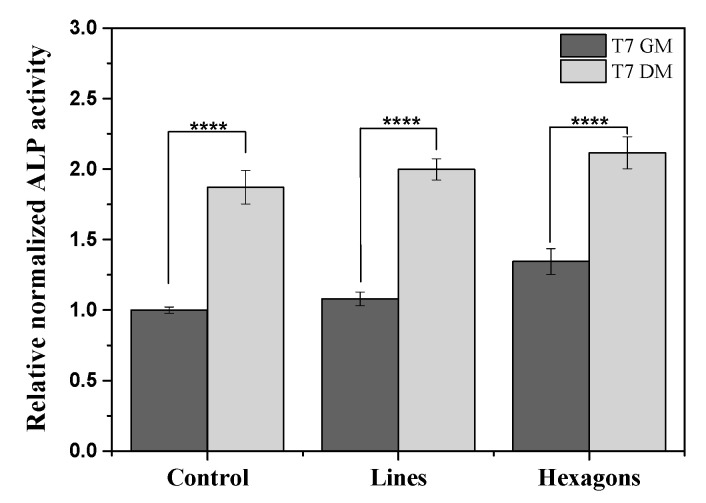
Osteogenic differentiation determined by relative alkaline phosphatase quantification assay expression after 7 days of culture (T7), using growth medium (GM) and differentiation medium (DM). The ALP expression was normalized against DNA content using CyQuant cell proliferation assay. **** *p* < 0.0001 (two-way ANOVA).

After 7 days, ALP was evaluated in the different samples. Alkaline phosphatase is an enzyme mostly found in liver, kidney, and bones, and the measurement of its activity has been found to be suitable for monitoring changes in bone formation and thus in bone cells differentiation [23]. It was found that ALP activity levels are significantly higher for cells under DM, compared to GM (Figure 2). The physical influence of hexagons topographies on the improvement of differentiation can be confirmed by cells’ ALP activity in 7 days of culture with GM, compared to cells over the control and lines patterned scaffolds. These results demonstrating the positive influence of the hexagonal topography are further supported by the alizarin red staining results.

**Figure 3:**
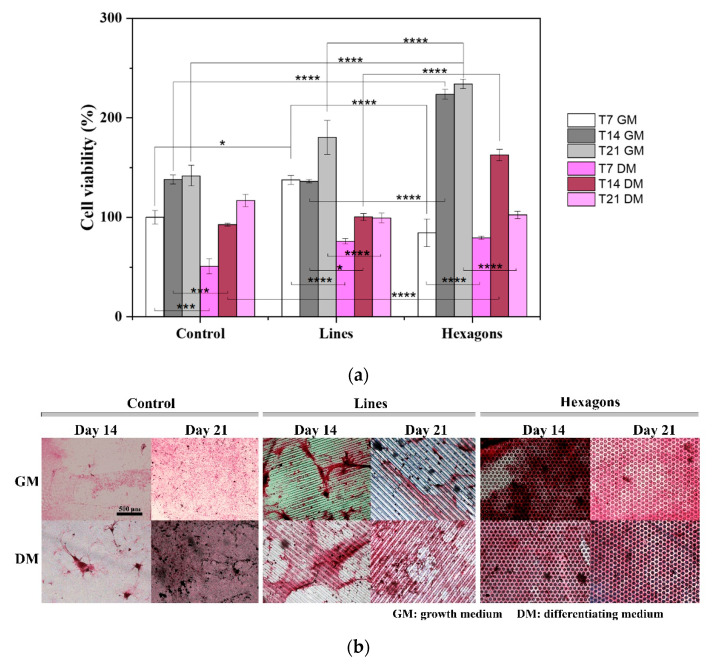
(**a**) Cellular viability of pre-osteoblast cells in contact with control, linear, and hexagonal scaffold topographies, in a differentiation essay of 7, 14, and 21 days, with GM and DM. * *p* < 0.05, *** *p* < 0.001, **** *p* < 0.0001 (two-way ANOVA); (**b**) Alizarin red staining for mineral deposition during osteogenesis induction, at day 14 and 21, with and without DM. Calcified areas are presented with pink color. The scale bar 500 µm is valid for all the images.

After 14 and 21 days of cell differentiation, the cell viability assay and alizarin red staining were performed (Figure 3).

Cell viability results (Figure 3a) were compared to a cell mineralization assay (Figure 3b). The presence of DM induces differentiating pathways on cells, providing chemical stimuli to stop proliferating and begin differentiation processes. On the contrary, basal GM provides cells with all the nutrients necessary to proliferate continuously until reaching the confluency. In DM conditions, cells present lower viability for all topographies and dimensions, compared to GM (Figure 3a). Complementing the viability results with the alizarin red images, in order to evaluate the mineralization of bone cells, a lower osteogenic differentiation of cells over linear topographies can be seen, which was predictable from the proliferation assays. On the other hand, the degree of mineralization on isotropic hexagon patterned scaffolds is very similar both with GM and DM after both 14 and 21 days (Figure 3b). These results indicate that the provided physical effect is able to regulate cell fate and may activate differentiation signaling pathways, with no need of biochemical inducers.

In this way, it is concluded that pre-osteoblast cells can be differentiated into osteoblast by specific patterns that also support matrix mineralization.

## 3. Materials and Methods

### 3.1. Materials

Poly(vinylidenefluoride-co-trifluoroethylene); P(VDF-TrFE), with 70 mol% vinylidene fluoride and 30 mol% trifluoroethylene, from Solvay (Póvoa de Santa Iria, Portugal); and N,N-dimethylformamide, DMF, from Merck (Sintra, Portugal), were used as received. An 8% (*w*/*w*) polymer solution in DMF was prepared under magnetic stirring at room temperature.

### 3.2. Samples Processing and Main Characteristics

Patterned scaffolds were obtained through photolithography and replica molding techniques, as described in [7,24]. In short, lines and hexagonal patterns with different dimensions (25, 75, and 150 µm) were designed in AutoCAD 2018 software and printed in photolithographic masks by Microlitho (see supplementary information—Figures S2 and S3). The photolithographic masks were used for the fabrication of SU-8 patterned molds, through photolithography, which were replicated in flexible and reusable polydimethylsiloxane (PDMS) molds, through replica molding. The P(VDF-TrFE) in DMF polymer solution was deposited on both patterned and non-patterned PDMS molds, previously treated with an oxygen plasma for 10 min, and evaporated at 100 °C [25]. The obtained samples present hydrophobic behavior (>100°) and a crystallinity degree of ≈ 32%, as shown previously [7].

### 3.3. Samples Sterilization

Circular samples with 6 mm diameter were cut, exposed to ultraviolet (UV) light on each side for 1 h, and then placed in standard 48-well cell culture plates. All samples were washed five times (5 min each time) with sterile phosphate buffer saline (PBS) 1× solution.

### 3.4. Cell Culture

MC3T3-E1 pre-osteoblasts (Riken bank, Tsukuba, Japan) were grown in a 75 cm^2^ cell-culture flask with modified Eagle’s medium (DMEM, Biochrom, Berlin, Germany) containing 1 g∙L^−1^ glucose, 1% penicillin/streptomycin (P/S, Biochrom, Berlin, Germany) and 10% Fetal Bovine Serum (FBS, Biochrom, Berlin, Germany). The flask was placed in a 37 °C incubator under 95% humidified air and 5% CO_2_ conditions. Culture medium was changed every two days and, at a 60–70% confluence, cells were trypsinized with 0.05% trypsin-EDTA (Biochrom, Berlin, Germany). A 25 μL drop of cell suspension was added over each P(VDF-TrFE) sample with a density of 10 × 10^4^ cells.mL^−1^ for the proliferation assays (cell viability) and at a density of 50 × 10^4^ cells∙mL^−1^ for the differentiation studies (cell viability, ALP and alizarin red). After that, the plates were incubated during 30 min for cell adhesion. The well volume was then completed with the growth medium (GM) and incubated once more for 24 h.

For proliferation assessment, cells were cultivated for 3 days, with 24 h and 72 h as timepoints. For the differentiation assays, the medium was exchanged by osteogenic differentiating medium (DM) after 24 h and cells were maintained up to 21 days, with 7, 14, and 21 days as timepoints. DM was composed of the GM supplemented with 0.1 µM dexamethasone (Sigma-Aldrich, Sintra, Portugal), 50 µg.mL^−1^ of ascorbic acid (Sigma-Aldrich, city, state, country), and 10 mM of b-glycerophosphate (Sigma-Aldrich, Sintra, Portugal). Culture media were changed every two days.

### 3.5. Cell Viability

Viable cells in proliferation and differentiation assays were quantified at the different timepoints using a CellTiter 96^®^ AQueous One Solution Cell Proliferation Assay (MTS, Promega, Madison, WI, USA). For that, the samples were incubated in an MTS:GM (1:5) solution for 3 h at 37 °C, under 95% humidified air and 5% CO_2_ conditions, and the absorbance at 490 nm was recorded with a microplate reader (Biotech Synergy HT, Winooski, VT, USA,). The quantitative results of proliferative cells will be presented as mean ± standard deviation (SD) and the results of the cells differentiation will be presented as mean ± standard error of mean (SEM), both of quadruplicated samples.

### 3.6. Immunofluorescence Staining

At the proliferation assay timepoints (24 and 72 h), two replicates of each sample were fixed with 4% formaldehyde (Panreac AppliChem, Barcelona, Spain) and subjected to immunofluorescence staining, to analyze their behavior in the different culture patterns. Cell’s cytoskeleton was stained with 1 μg.mL^−1^ of phalloidin tetramethylrhodamine (TRITC, Sigma Aldrich, Sintra, Portugal) solution for 45 min at room temperature, and cells’ nucleus with 1 μg.mL^−1^ of a 4,6-diamidino-2-phenylindole (DAPI, Sigma Aldrich, Sintra, Portugal) solution for 5 min. Samples were washed with PBS 1× before, after, and during the steps. Finally, the samples were visualized with fluorescence microscopy (Olympus BX51 Microscope, Lisboa, Portugal).

### 3.7. Quantification of DNA and Alkaline Phosphatase Activity

Cells’ DNA content was measured using a CyQUANT^®^ Cell Proliferation Assay Kit (Life Technologies, Porto, Portugal) and the osteogenic capacity was determined through an ALP (Sigma Aldrich, Sintra, Portugal), both of which are described in [26]. Briefly, after 7 days of cell culture on both GM and DM, cells were lysed with Triton 0.1% buffer and frozen at 70 °C. After thawing, 50 μL *p*-nitrophenyl phosphate and 50 μL 2-amino-2-methyl-1-propanol were added, according to manufacturer’s protocol. Using a microplate reader, the amount of the produced *p*-NP (*p*-nitrophenol) was measured, reading the absorbance at 405 nm. To proceed to the ALP activity normalization, cells’ DNA content was quantified from the cell lysate using the CyQUANT^®^ Cell Proliferation Assay Kit, according to the manufacturer’s protocol, measuring its fluorescence by exciting the sample at 480 nm and measuring the emission at 520 nm, using the same microplate reader. The results will be presented as mean ± SEM of quadriplicated samples.

### 3.8. Mineralization Assay

At the differentiation timepoints (7, 14, and 21 days), the samples were marked with 10% alizarin red solution in acetic acid, allowing the qualitative detection of calcium deposits, characteristic of osteogenic differentiation.

### 3.9. Statistical Analysis

Statistical analysis was carried out by ANOVA using Tukey test (Graphpad Prism 8, San Diego, CA, USA), with *p* values < 0.05 considered to be statistically significant.

## 4. Conclusions

This study reports on the influence of different patterned piezoelectric P(VDF-TrFE) scaffolds in the preosteoblasts’ proliferation and differentiation. It was observed that MC3T3-E1 cells differentiation can be induced solely by a physical stimulus, specifically by an isotropic hexagonal surface topography, with no need of using a chemical inducer. Finding that bone cells differentiation can be positively influenced by this type of geometry, it is concluded that novel scaffolds based on electromechanically active materials with specific geometries can provide the necessary geometrical and electroactive components of the bone microenvironment to support bone regeneration.

## Supplementary Materials

Supplementary Materials can be found at https://www.mdpi.com/1422-0067/21/21/8352/s1. Figure S1. FTIR-ATR spectra of P(VDF-TrFE) scaffolds. Figure S2. Schematic representation of the produced patterned P(VDF-TrFE) scaffolds with linear microstructures. Figure S3. Schematic representation of the patterned P(VDF-TrFE) scaffolds with hexagonal microstructures. Figure S4. Cell adhesion of MC3T3-E1 on dense non-patterned P(VDF-TrFE) scaffolds.
