# Supplementary material for: Patterned Piezoelectric Scaffolds for Osteogenic Differentiation
Source: Int J Mol Sci. 2020 Nov 7;21(21):8352. doi: 10.3390/ijms21218352 (PMC7672637; doi:10.3390/ijms21218352)
Supplement: Supplementary file 1 [file ijms-21-08352-s001.pdf]

Supplementary Materials

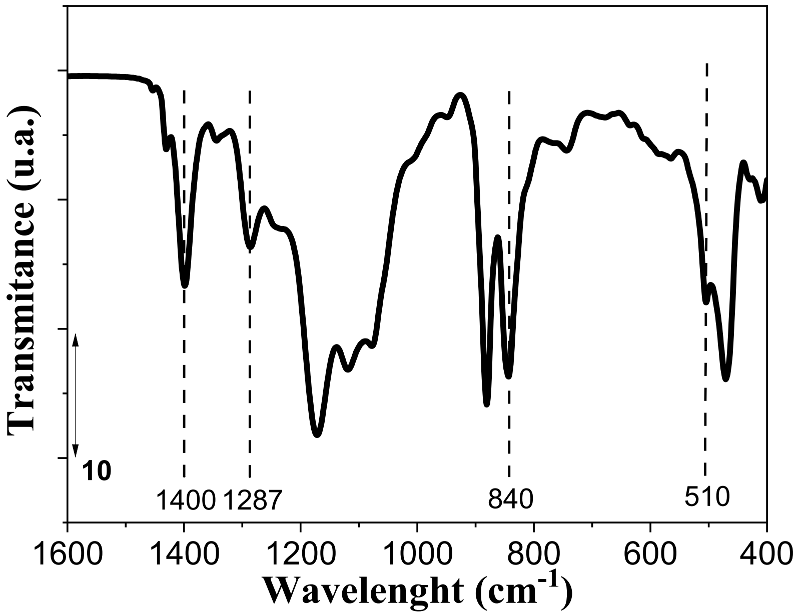

Figure S1. FTIR-ATR spectra of P(VDF-TrFE) scaffolds.

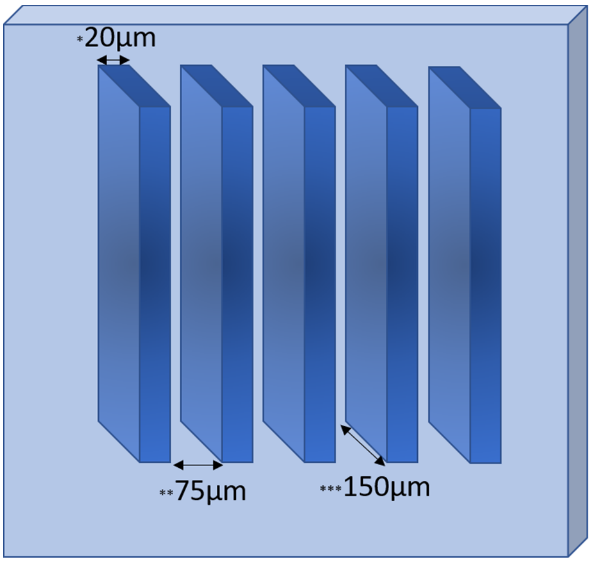

Figure S2. Schematic representation of the produced patterned P(VDF-TrFE) scaffolds with linear microstructures.

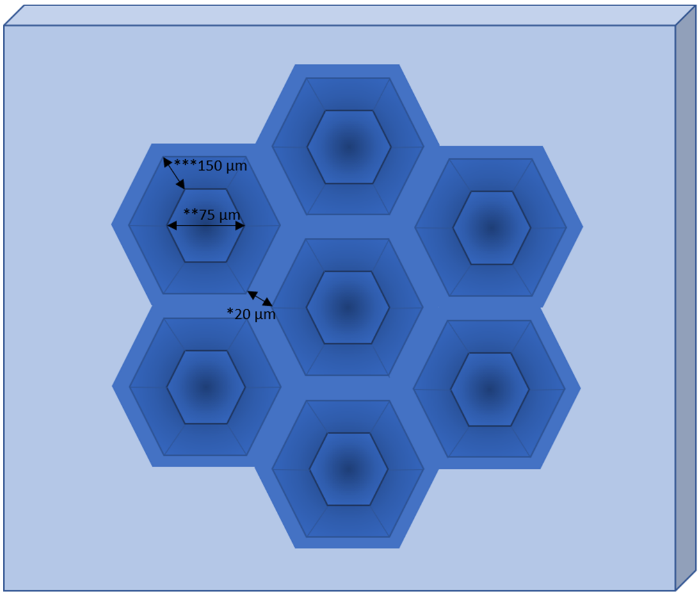

Figure S3. Schematic representation of the patterned P(VDF-TrFE) scaffolds with hexagonal microstructures.

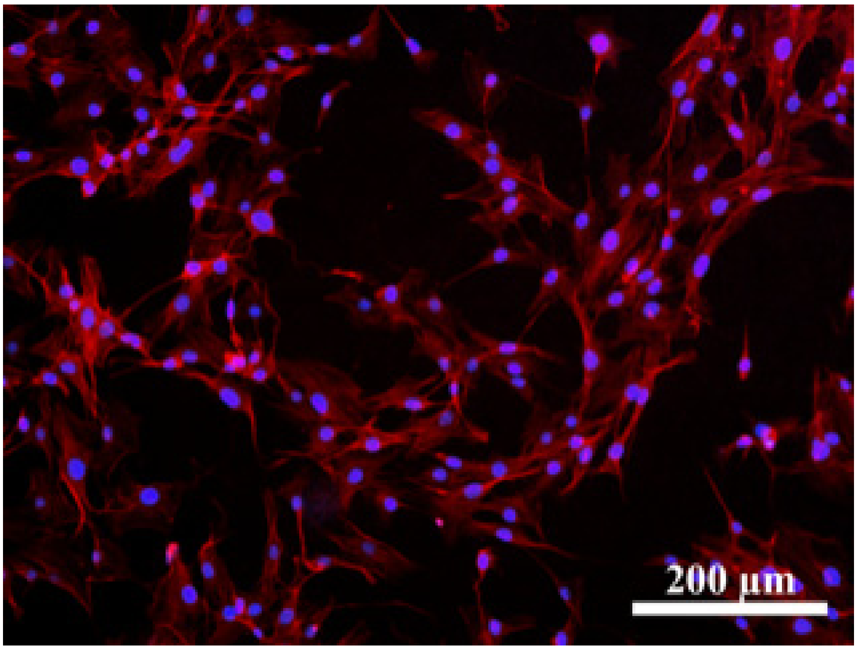

Figure S4. Cell adhesion of MC3T3-E1 on dense non-patterned P(VDF-TrFE) scaffolds.
